# Porous silicon Bloch surface and sub-surface wave structure for simultaneous detection of small and large molecules

**DOI:** 10.1186/1556-276X-9-383

**Published:** 2014-08-07

**Authors:** Gilberto A Rodriguez, John D Lonai, Raymond L Mernaugh, Sharon M Weiss

**Affiliations:** 1 Department of Electrical Engineering and Computer Science, Vanderbilt University, Nashville, TN 37235, USA; 2 Department of Physics, Northwest Nazarene University, Nampa, ID 83686, USA; 3 Department of Biochemistry, Vanderbilt University, Nashville, TN 37232, USA

**Keywords:** Bloch surface wave, Bloch sub-surface wave, Size selective, Large molecule, Biosensor, M13KO7 bacteriophage, Nanospheres

## Abstract

A porous silicon (PSi) Bloch surface wave (BSW) and Bloch sub-surface wave (BSSW) composite biosensor is designed and used for the size-selective detection of both small and large molecules. The BSW/BSSW structure consists of a periodic stack of high and low refractive index PSi layers and a reduced optical thickness surface layer that gives rise to a BSW with an evanescent tail that extends above the surface to enable the detection of large surface-bound molecules. Small molecules were detected in the sensor by the BSSW, which is a large electric field intensity spatially localized to a desired region of the Bragg mirror and is generated by the implementation of a step or gradient refractive index profile within the Bragg mirror. The step and gradient BSW/BSSW sensors are designed to maximize both resonance reflectance intensity and sensitivity to large molecules. Size-selective detection of large molecules including latex nanospheres and the M13KO7 bacteriophage as well as small chemical linker molecules is reported.

## Background

Porous silicon (PSi) has excelled as a biosensing platform due to its cost-effective and versatile fabrication, enhanced surface area, and chemical and biological compatibility. Well-established Si surface functionalization chemistry has led to specific binding of several relevant molecules including DNA [1], proteins [2], explosives [3], and illicit drugs [4] to PSi platforms. However, PSi refractometric sensing applications have generally been size limited to molecules that diffuse into the porous matrix to cause a measurable change in effective optical thickness. Pore sizes of 5 to 100 nm diameter have allowed for the detection of larger molecules such as bovine serum albumin (8 nm in width) and anti-MS2 antibodies (15 nm in width) [5,6]. However, molecules approaching the average pore diameter clog the pore and hinder molecular infiltration, which significantly deteriorates the transduced signal. For example, 40-base DNA (~13 nm in length) cannot efficiently infiltrate 20-nm pores [7,8]. Hence, there is a significant challenge in detecting biological entities such as viruses, bacteria, and blood cells that typically have sizes much larger than those of the pores. Alternative measurement techniques for the detection of surface-bound molecules on PSi include monitoring fluorescent labels and changes in reflectance intensity for the detection of MS2 bacteriophage [6] and *Escherichia coli* bacteria [9], respectively. However, emerging interest in lab-on-a-chip technologies has placed focus on label-free refractometric-based sensors in order to avoid the additional expense of fluorescent labels. In addition, refractometric sensing configurations are a popular choice due to the compact size, small active sensing region, ability to transduce molecular interaction with an electric field into a refractive index change, and ability to array and multiplex devices allowing several biosensors on a single chip. For example, silicon-on-insulator (SOI) waveguides (WGs) and surface plasmon devices utilize evanescent fields to detect surface-bound molecules of all sizes [10,11]. PSi WGs have demonstrated sensitivities an order of magnitude greater than SOI WGs due to the direct interaction of small molecules with the guided field inside the porous layer; however, surface-bound large molecules present a detection challenge in PSi WGs due to the weak evanescent fields at the surface [8,12,13]. The PSi BSW/BSSW biosensor offers the possibility to detect both small molecules that infiltrate the pores and large molecules attached to the sensor's surface [8]. The BSW mode is a surface state excited within the truncated defect layer at the surface of a multilayer Bragg mirror and has been previously reported in PSi sensing applications [14-17]. The novel BSSW mode is confined by a step or gradient refractive index within the multilayer and can selectively detect small molecules attached within the pores with an enhanced sensitivity (>2,000 nm/refractive index unit (RIU)) in comparison to band edge modes of the multilayer, microcavities, or traditional WG modes [8,12,16]. The BSW and BSSW modes are each manifested as a distinct resonance peak in the reflectance spectrum, and the angular shift of each peak can be used to quantify the number of molecules attached to the sensor. A thorough theoretical analysis of both the step and gradient BSW/BSSW configurations has been previously presented [8]. In this report, the first fabricated step index and an optimized gradient index PSi BSW/BSSW biosensor are presented. Large M13KO7 bacterial viruses and 60 nm diameter latex nanospheres as well as small 3-aminopropyltriethoxysilane (APTES) and gluteraldehyde (GA) molecules are used as model systems to demonstrate the size-selective detection scheme.

## Methods

### PSi BSW/BSSW device fabrication

**Figure 1:**
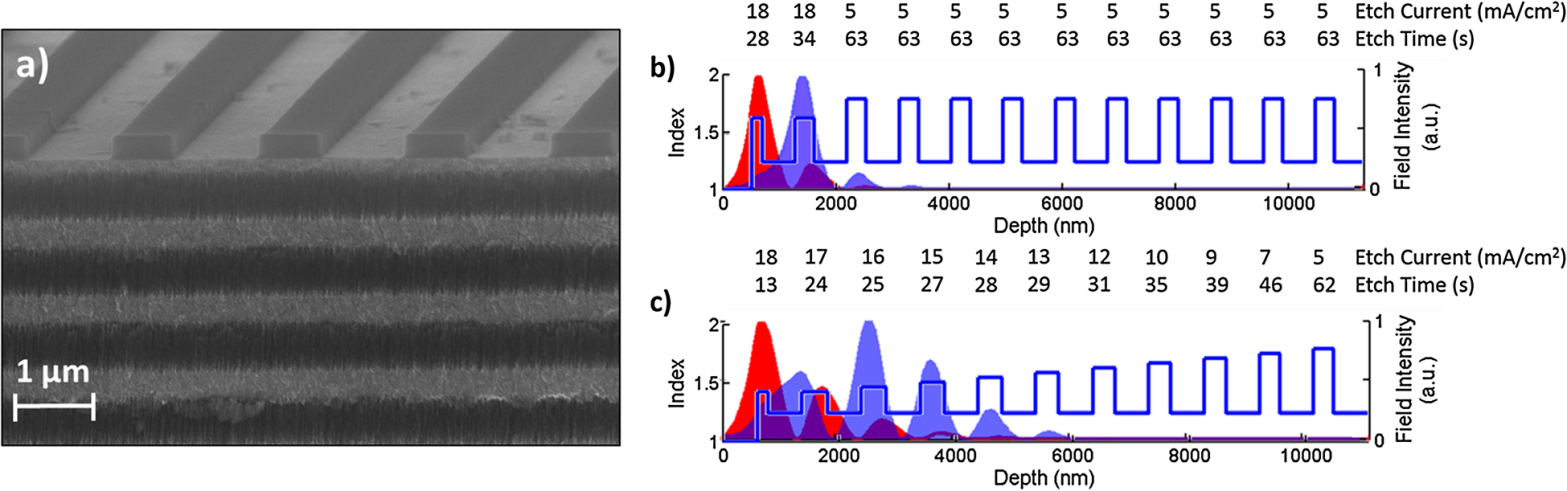
**SEM image and etch parameters of PSi BSW/BSSW sensor. (a)** SEM cross-sectional image of PSi BSW/BSSW sensor. Refractive index profiles of **(b)** step and **(c)** gradient index BSW/BSSW sensors where the numbers shown above each layer represent the etch current (mA/cm^2^) and etch time (s), respectively. The field intensity of the BSW mode (red) and 1st BSSW modes (blue) are shown within the corresponding layers of the sensor.

In this work, the step and gradient refractive index profiles are created during the electrochemical etching of p + (approximately 0.01 Ω cm) Si (100) in a 15% hydrofluoric acid solution. The number of periods of the multilayer and the depth of the step and gradient refractive index layers were determined based on transfer matrix and rigorous coupled wave analysis (RCWA) simulations as explained in the ‘Results and discussion’ section. The BSW/BSSW multilayer contains periods of alternating high (H) and low (L) refractive index layers with the first layer being truncated as shown in the cross-sectional scanning electron microscope (SEM) image in Figure 1a. Etch parameters for each H layer of the step and gradient index profiles are described in Figure 1b,c, respectively, where the top number is the current density in mA/cm^2^ and the bottom number is the etching duration in seconds. All L layers are etched with a 48 mA/cm^2^ current density for 22 s. The samples are then placed in a 1.5 mM l^−1^ potassium hydroxide in ethanol solution for 5 min and oxidized for 5 min at 500°C in air. Gratings of pitch 1,820 and 1,650 nm are patterned onto the gradient and step index BSW/BSSW structures, respectively, via electron beam lithography on a 250-nm-thick ZEP 520A photoresist. The indices and thicknesses shown in Figure 1b,c were determined after fabrication through SEM images and by matching measured angular reflectance spectra with RCWA simulations.

### Latex nanosphere functionalization

**Figure 2:**
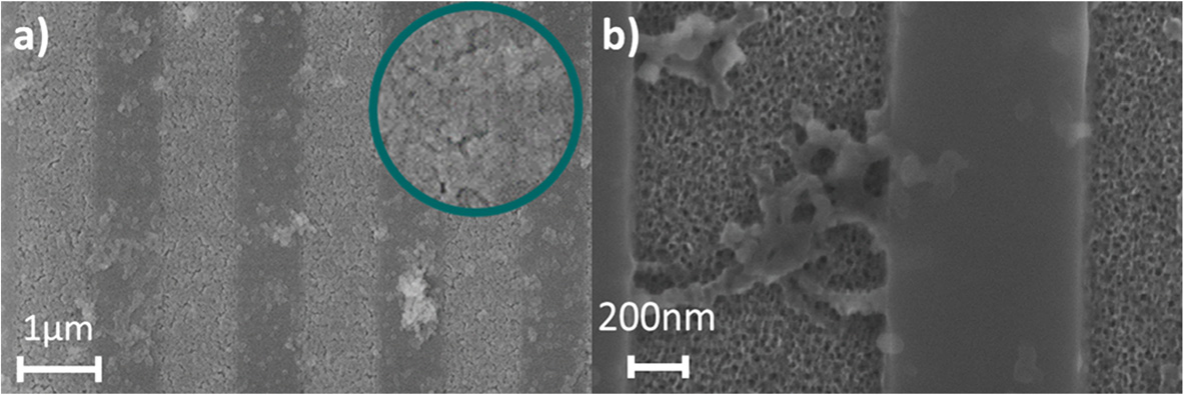
Top view SEM images of (a) 60-nm latex nanosphere and (b) M13KO7 bacteriophage attachment to the PSi BSW/BSSW surface.

Size-selective molecular detection was demonstrated using a prototypical small chemical molecule, APTES (size ≈ 0.8 nm), and large, 60-nm carboxyl latex nanospheres. A 4% APTES solution was prepared in methanol and water, and an aliquot was placed on the PSi sample for 10 min. The sample was subsequently immersed in methanol for 10 min to rinse away excess APTES molecules not attached to the PSi and then thermally annealed for 10 min at 150°C. The sample was then rinsed with methanol to remove any remaining unbound APTES molecules. A 4% *w*/*v* solution of carboxyl terminated latex nanospheres (Invitrogen™, Thermo Fisher Scientific, Carlsbad, CA, USA) was placed on the BSW/BSSW sensor for 1 min followed by a thorough methanol and deionized (DI) water rinsing. Attachment and quantification of the small and large species were determined by monitoring the angle-resolved reflectance spectrum in between molecular attachments. The attachment of the nanospheres was additionally verified by SEM imaging as shown in Figure 2a. No spheres were observed to penetrate the porous matrix in cross-sectional images (not shown).

### M13KO7 bacteriophage functionalization

Viruses are infectious agents that can cause disease in humans, plants, and animals; antibodies are typically used in immunoassays to detect viruses in biological samples. The M13KO7 bacterial virus was used as a model system to determine if the large (approximately 2 μm in length; 16,400 kDa) M13KO7 could be directly bound to and detected on the PSi BSW/BSSW sensor surface. The M13KO7 bacteriophage is a low-cost, readily available, nonhazardous *E. coli* bacterial virus that can be readily detected using commercially available antibodies [18,19]. The virus was covalently cross-linked to the PSi surface via APTES and GA linkers. APTES was attached as described above. GA is a homobifunctional cross-linker that can bind to and covalently link molecules through their free amines. A 2.5% GA in phosphate buffered saline (PBS) buffer solution was used to cross-link the APTES free amines on the sensor surface to the free amines on M13KO7 suspended in solution on the sensor surface. After a 30-min GA incubation step, a 1% sodium cyanoborohydride (Sigma-Aldrich, St. Louis, MO, USA) in PBS buffer solution was applied, followed by a 30-min incubation step to stabilize the Schiff base bonds formed during GA cross-linking [20]. The M13KO7 (0.32 mg/ml carbonate/bicarbonate buffer, pH ~ 10) was diluted to a final concentration of 32 μg/ml in PBS buffer (final pH ~ 9.5) and applied to the sensor surface for 20 min at room temperature. The device was thoroughly rinsed with DI water. Figure 2b shows a top view SEM image of the M13KO7 bacteriophage immobilized on the PSi surface. Coulombic interactions prevent a uniform self-assembled monolayer due to the negatively charged nature of the virus.

## Results and discussion

**Figure 3:**
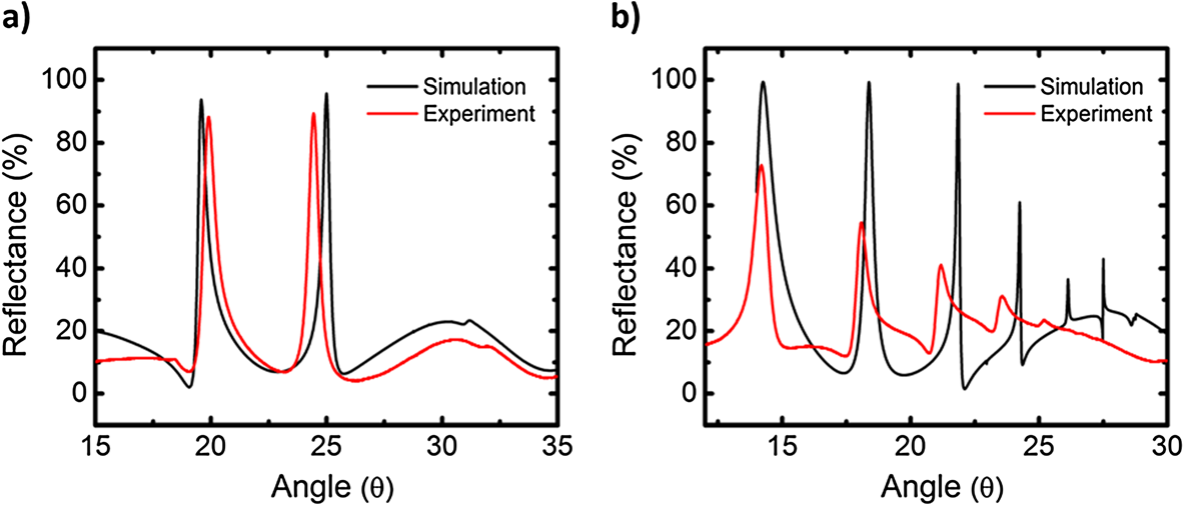
**Simulated and experimental reflectance spectra of optimized (a) step and (b) gradient index PSi BSW/BSSW sensor in air.** The resonance at the lowest angle for each sensor corresponds to the BSW mode while the other resonances are BSSW modes. Simulations show good agreement with experiment, with small error derived from nonlinear refractive index changes within the PSi multilayer.

A resonance condition is distinctly excited when the effective index of a BSW or BSSW mode is matched by the coupling conditions of either a prism or diffraction grating. Prism coupling is compatible with existing surface plasmon resonance biosensing instrumentation. Grating coupling allows for more compact devices, which could be used for point of care diagnostics with microfluidics integration [21]. The BSW mode is confined by the band gap created by the Bragg mirror and by total internal reflection near the surface. Similarly, by reducing the optical thickness of one or more layers within the multilayer through the introduction of a step or gradient refractive index profile, BSSW modes with different effective indices can be supported within the multilayer. The implementation of a single step to break the periodicity of the Bragg mirror refractive index profile shifts the band edge of the Bragg mirror and gives rise to a single BSSW mode confined within the corresponding layer with reduced optical thickness. By creating a gradient refractive index profile, a varying band edge within the multilayer allows a distinct mode within each H layer until prohibited by coupling losses or a band gap is no longer sustained [8]. Although a large sensitivity is important in biosensor design, a sharp and distinct resonance will enhance the minimum detectable shift for an improved detection limit. Therefore, in the design of the step and gradient profile structures, a tradeoff between sensitivity of the resonance position to small changes in refractive index and the resonance intensity was considered. A very small step or gradient refractive index change leads to a very large BSSW sensitivity. However, similar to a WG, the resonance intensity and mode confinement are reduced with a small refractive index contrast between H and L layers due to the reduced mirror strength of the multilayer. For very large refractive index changes within the multilayer, field confinement is increased, resulting in a sharp and distinct resonance; however, BSSW sensitivity decreases as a result of decreased surface area for molecular capture [22]. Figure 3 shows both the simulated (RCWA) and experimental angle-resolved reflectance spectra of an optimized grating-coupled step and gradient index BSW/BSSW sensor. In Figure 3a, the BSW resonance is located at approximately 21° and the single step BSSW mode is located at approximately 25°. In Figure 3b, the BSW mode is located at approximately 15° and the remaining peaks correspond to the different BSSW orders created by the gradient index profile. The different resonance angles are a result of the different refractive index step and gradient depth profiles used in the optimization. Good agreement is observed between the simulations and experiment. Minor deviations are likely a result of a nonlinear refractive index gradient or step caused by the KOH etch [8]. Both the step and gradient BSW/BSSW designs are suitable for size-selective sensing applications. However, the step index sensor has a higher detection sensitivity due to the single well-confined BSSW resonance, as shown in the field profile in Figure 1b, while the gradient index sensor with multiple BSSW modes spatially distributed within several high index layers of the multilayer allows for the determination of the depth of infiltration of molecules within the multilayer.

**Figure 4:**
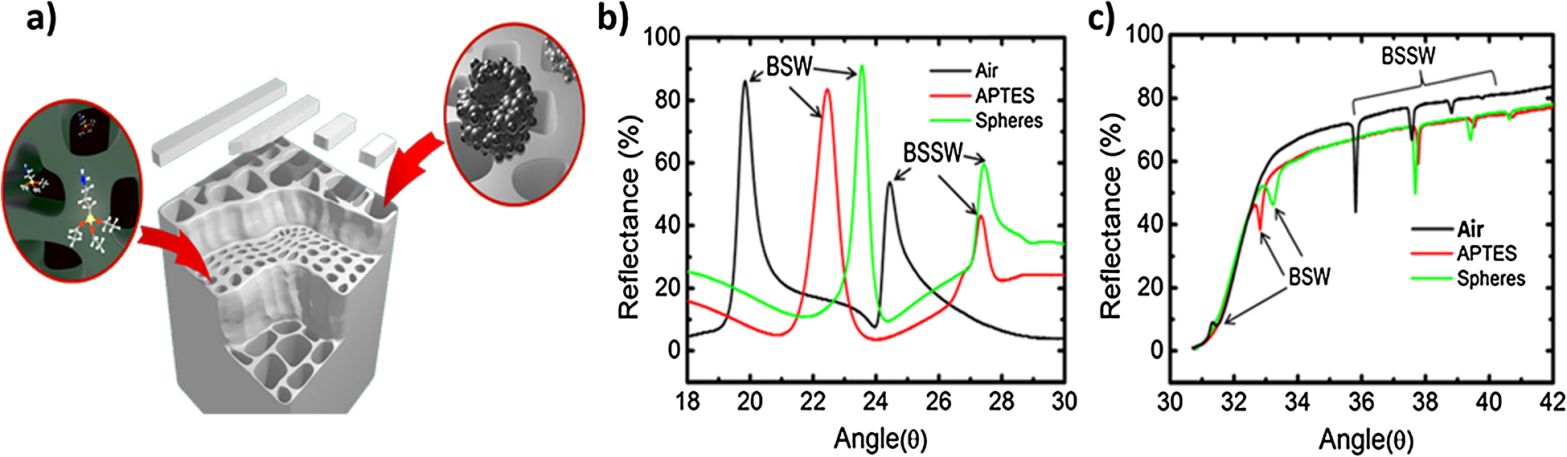
**Schematic of size-selective sensing experiment and reflectance spectra of BSW/BSSW sensors after APTES and large latex sphere attachment. (a)** Schematic illustration of size-selective sensing experiment in which both small and large species are exposed to the PSi BSW/BSSW sensor. Reflectance spectra of **(b)** grating-coupled step index and **(c)** prism-coupled gradient index BSW/BSSW sensors after exposure to small (APTES) and large (spheres) species. The BSW resonance and BSSW resonances are labeled in the figure. The BSW resonance in each sensor shifts to higher angle when APTES and spheres are attached to the respective sensor but the BSSW resonances only shift with APTES attachment because the BSSW modes do not extend above the sensor surface where the spheres are bound. Table 1 summarizes the resonance shifts shown in this figure.

In order to demonstrate the sensing capabilities of the step and gradient index BSW/BSSW, small APTES molecules that bind primarily within the porous matrix and large nanospheres that may only bind onto the surface of the PSi are exposed to the sensors (Figure 4a). Results of the attachments of APTES and nanospheres to the step (grating-coupling configuration) and gradient (prism-coupling configuration) index sensors are depicted in Figure 4b,c, respectively. The step index profile gives rise to a single BSSW mode while the gradient index profile has three BSSW modes due to the varying band edge within the multilayer. The spectrum label ‘air’ was measured after oxidation of the BSW/BSSW structure. The subsequent attachment of APTES and then nanospheres leads to a redshift in the spectrum corresponding to the addition of material to the sensor. When APTES is attached, both the BSW and BSSW(s) modes shift in resonance positions due to APTES infiltrating all regions where the fields are confined. When the nanospheres are attached to the APTES functionalized sensor, they cannot penetrate the layers where the BSSW field is primarily confined and therefore do not cause a significant shift to the BSSW resonance position. The evanescent field of the BSW is able to detect the presence of nanospheres in both the prism- and grating-coupled configurations. Table 1 contains the angular resonance shift data of the step and gradient index BSW and 1st order BSSW shown in Figure 4. The 2nd and 3rd order BSSWs of the gradient index BSW/BSSW sensor demonstrate a detection sensitivity that is similar to that of the 1st order BSSW. The larger sensitivity of the step index sensor compared to the gradient index sensor derives from the smaller refractive index step depth as shown in Figure 1a,b. Additionally, higher sensitivity in grating-coupled structures has been observed in comparison to prism-coupled structures [23]. The slight blue shift reported for the gradient index BSSW after exposure to the nanospheres may be attributed to loss of a small fraction of the immobilized APTES molecules that could occur during the rinsing stage as a result of APTES molecules' compromised stability in water [24].

**Table 1:** **Resonance shifts illustrated in Figure**4

**Molecules**	**BSW (step)**	**BSSW (step)**	**BSW (gradient)**	**BSSW (gradient)**
APTES	2.60°	2.88°	1.32°	1.96°
Spheres	1.11°	0.25°	0.42°	−0.08°

**Figure 5:**
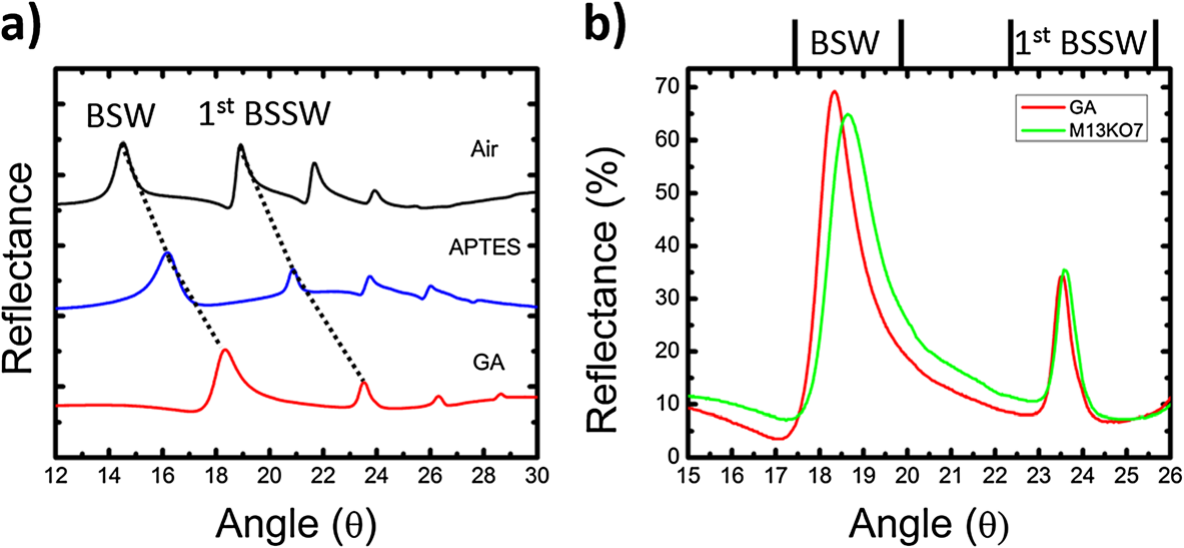
**Reflectance spectra illustrating resonance shifts of the BSW/BSSW modes caused by small linker molecules and the M13KO7 bacteriophage. (a)** Angular reflectance spectra of an oxidized gradient index BSW/BSSW sensor measured before (black) and after the attachment of APTES (blue) and GA (red). The spectra are offset for clarity. The lowest angle resonance on each plot corresponds to the BSW mode. Three BSSW resonances appear at higher angles. **(b)** Resonance shifts of the BSW and 1st BSSW mode after the attachment of M13KO7 bacteriophage to the GA functionalized gradient index BSW/BSSW sensor shown in **(a)**. Quantification of the angular shifts is reported in the text.

In order to demonstrate the feasibility for detecting large biological organisms that have previously presented a challenge in PSi sensors, the M13KO7 bacteriophage was immobilized onto a grating-coupled gradient index BSW/BSSW sensor. APTES and GA are small chemical linker molecules that infiltrate the pores and are therefore detected by both the BSW and BSSW modes as shown in Figure 5a. Resonance shifts for APTES and GA for the BSW and 1st BSSW mode are (1.6°; 2.18°) and (1.97°; 2.66°), respectively. The large M13KO7 bacteriophage does not infiltrate the 20-nm pores and is solely detected by the BSW with a resonance shift of 0.31° (Figure 5b). The BSSW shows a small shift of 0.01° that can be attributed to the small evanescent field of the BSSW at the surface (Figure 1c). In future applications, the M13KO7 virus can be selectively bound to the surface using an antibody probe method similar to that reported in [6]. The response of the BSW to the model virus leads to the conclusion that the BSW mode is able to monitor changes in refractive index to detect large organisms such as cells, bacteria, and viruses that are selectively bound to the surface using appropriate chemical functionalization. The BSW/BSSW is a versatile sensor with possible integrations with lab-on-a-chip technology to detect small molecules with an extremely high sensitivity (>2,000 nm/RIU) and will not be limited in detecting large species that cannot infiltrate the pores.

## Conclusions

The fabrication and realization of step and gradient index BSW/BSSW sensors were demonstrated. The excitation of both BSW and BSSW modes within the same structure in both grating- and prism-coupled configurations allowed for simultaneous detection of APTES and GA with both modes and the detection of large 60-nm nanospheres and the large M13KO7 bacteriophage with the BSW. The strong confinement of the BSSW minimizes the overlap with surface immobilized analytes for high sensitivity, high selectivity applications. The evanescent field of the BSW allows for detection of very large molecules that could not be detected in typical PSi devices such as interferometers, microcavities, and waveguides. Size-selective detection using the same sensor platform is expected to be a significant advantage for future multianalyte detection schemes using a microfluidics approach.

## Abbreviations

APTES: 3-aminopropyltriethoxysilane; BSW: Bloch surface wave; BSSW: Bloch sub-surface wave; GA: gluteraldehyde; H: high refractive index; L: low refractive index; PBS: phosphate buffer saline; PSi: porous silicon; RCWA: rigorous coupled wave analysis; SEM: scanning electron microscope; SOI: silicon-on-insulator; WG: waveguide.

## Competing interests

The authors declare that they have no competing interests.

## Authors’ contributions

GAR led the experimental and computational efforts on the BSW/BSSW sensors. JDL assisted in optimizing the BSW/BSSW structures and conducted initial nanosphere experiments. RLM recommended M13KO7 bacteriophage as a large model virus for detection on PSi and assisted in developing chemical immobilization methods. SMW contributed to the design and analysis of the BSW/BSSW experiments. All authors read and approved the final manuscript.
